# Autoimmune-induced preferential depletion of myelin-associated glycoprotein (MAG) is genetically regulated in relapsing EAE (B6 × SJL) F1 mice

**DOI:** 10.1186/1750-1326-3-7

**Published:** 2008-06-09

**Authors:** Dusanka S Skundric, Rujuan Dai, Vaagn L Zakarian, Weili Zhou

**Affiliations:** 1 Department of Neurology Wayne State University School of Medicine, Detroit, USA; 2 Department of Immunology and Microbiology, Wayne State University School of Medicine, Detroit, USA; 3 Department of Computer Science, University of Colorado, Denver, USA

## Abstract

**Background:**

Experimental autoimmune encephalomyelitis (EAE) is commonly used to investigate mechanisms of autoimmune-mediated damage to oligodendrocytes, myelin, and axons in multiple sclerosis (MS). Four distinct autoimmune mechanisms with subsequently distinct patterns of demyelination have been recognized in acute MS lesions. EAE correlates for those distinct patterns of MS lesions are unknown. An excessive loss of myelin-associated glycoprotein (MAG), as a result of distal oligodendrogliopathy, is found exclusively in the subtype III lesion. We sought to answer if types of demyelination in acute lesions during onset and relapse of EAE can replicate the specific patterns observed in MS acute lesions.

**Methods:**

In parental H-2^b ^(C57BL/6, B6) and hybrid H-2^b/s ^[(B6 × SJL) F1] EAE mice, we examined spinal cord levels of MOG, MAG, and myelin basic protein (MBP), and compared to levels of axonal neurofilament (NF160) to assess axonal function, and levels of PARPp85 as an indicator of irreversible apoptosis.

**Results:**

During disease onset, levels of MOG significantly dropped in both strains, although more profoundly in H-2^b/s ^mice. Levels of MOG recovered in relapsing mice of both strains. Regulation of MAG was dissimilar to MOG. Modest loss of MAG was found at disease onset in both strains of mice. Unexpectedly, in relapsing H-2^b/s ^mice, a major depletion of MAG and NF160, accompanied with sharp elevation of PARPp85 levels, was measured. PARPp85 immunoreactivity was observed in cytoplasm and nuclei of some MBP containing cells.

**Conclusion:**

Taken together, our results show genetically controlled distinct patterns of MOG and MAG depletion, in MOG_35–55 _induced EAE in H-2^b ^and H-2^b/s ^mice. The data also suggest distinctive immune regulation of acute lesions that develop in relapsing compared to disease onset. A profound depletion of MAG, concomitant with marked depletion of axonal NF160, and sharp elevation of PARPp85 levels, occurred exclusively in relapsing H-2^b/s ^mice. Our findings suggest concurrence of sharp decrease of MAG levels, axonal dysfunction and irreversible apoptosis with severe relapsing disease in H-2^b/s ^mice. We propose that MOG-induced EAE in H-2^b/s ^mice may prove as a useful model in studying mechanisms, which govern autoimmune-induced preferential loss of MAG, and its impact on oligodendroglial pathology.

## Background

Multiple sclerosis (MS) is debilitating neurological disorder with unknown etiology. Genetic control of susceptibility to MS has been linked to different DR and DQ loci within MHC class II. In patients with MS, autoimmune mediated inflammation, demyelination and axonal damage are commonly found within CNS lesions.

Experimental autoimmune encephalomyelitis (EAE) is an animal model, extensively used to gain better understanding into mechanisms of autoimmune mediated inflammation, demyelination and axonal damage. In mice, susceptibility to EAE is determined by H-2 restricted autoimmune responses to immunodominant epitopes of myelin proteins. This genetic control of autoimmune-induced encephalitogenic responses impact clinical symptoms, immunopathology and histopathology of EAE, and its similarities with the clinical course and histopathology of MS. Subsequently four distinct patterns, suggestive of different, specific underlying immune regulatory mechanisms have been proposed in MS [1]. Prominent changes typically observed in type III demyelination include signs of distal oligodendrogliopathy. An initial indicator of oligodendrocyte specific damage is depletion of myelin associated glycoprotein (MAG), which is a type I transmembrane glycoprotein positioned at periaxonal regions in the inner most loop of the oligodendrocyte cell processes. Specific loss of MAG is suggestive of oligodendroglial dysfunction and/or their death by apoptosis [2]. Because of the indispensable role of oligodendrocytes in myelin and axonal homeostasis, mechanisms of oligodendrocyte apoptosis remain among focal points in understanding immunopathogenesis of MS [3]. In both human disease and EAE, presence of apoptotic oligodendrocytes, but also infiltrating mononuclear cells, which undergo activation-induced cell death, has been documented. Subsequently, changes in levels of several pro- and anti-apoptotic regulatory molecules, including activation of terminal caspases, have been observed in MS and some models of EAE [4]. In this study we measured levels of PARPp85, which is an 85 kD caspase cleaved fragment of a DNA binding enzyme, poly (ADP-ribose) polymerase (PARP), and whose presence indicates irreversible apoptosis. In its cleaved form, PARP is insufficient in supporting DNA repair by itself and by other DNA binding enzymes.

Inflammatory demyelination initiates axonal dysfunction and/or loss, especially in relapsing-remitting MS. Damage and subsequent loss of axonal cytoskeletal neurofilaments correlates directly with neurological disability in MS [5]. The medium chain of neurofilament (NF160) is essential for myelination-dependent outside-in signaling, which arises from oligodendrocytes and controls axonal caliber and conduction velocity of motor axons [6,7].

As opposed to the extensively studied histopathology of heterogeneous lesions in human disease, it is largely unknown if any of the existing mouse EAE models can recapitulate specific patterns of demyelination found in MS. In order to use appropriate models to study specific pathogenetic mechanisms and to develop more specific therapies for MS, it is necessary to recognize specific and distinct demyelinating patterns in EAE.

In this study, we sought to answer the question if autoimmune demyelination directed towards an immunodominant epitope of myelin oligodendrocyte glycoprotein (MOG_35–55_), differs between two genetically related susceptible strains of mice. In addition, if proved that distinct types of demyelination are genetically determined in mice, do these patterns share some resemblance with types of lesions described in MS? In addressing those questions, we were aided by our previously described model of MOG_35–55 _induced EAE in (B6 × SJL) F1 (H-2^b/s^) mice, and by comparison to similarly induced EAE in C57BL/6 (B6) mice [8,9]. These two EAE models were advantageous for our studies because of the genetic relatedness of the parental H-2^b ^and hybrid H-2^b/s ^mice, and their susceptibility to H-2^b ^restricted MOG_35–55_. In addition, we and others reported that H-2^b ^and H-2^b/s ^mice mount distinct autoimmune responses to MOG_35–55_, which include differences in severity, incidence of relapse, extent and distribution of demyelinating lesions, immunopathology and other parameters related to antibody and T cell autoimmune responses [8,10].

In this study, we examined changes in relative levels of myelin proteins, which included MOG, myelin associated glycoprotein (MAG), and myelin basic protein (MBP) in spinal cord tissue throughout disease, in both strains of mice. In addition, we measured levels of medium chain of axonal neurofilament (NF160), and of caspase-cleaved fragment of poly (ADP-ribose) polymerase, PARPp80, in spinal cord of EAE mice.

We found preferential, marked depletion of MAG, decrease of NF160, and sharp elevation of PARPp80 levels only in relapsing H-2^b/s ^mice. Compared to H-2^b^, H-2^b/s ^mice developed more frequent and more severe relapses. Conversely, relative levels of MOG were reduced during acute disease in both strains, compared to control values. Loss of MOG in H-2^b/s ^markedly exceeded that in H-2^b ^mice. Levels of MBP were moderately decreased only in relapsing and chronic EAE in H-2^b/s ^mice. Overall, our data show genetically controlled distinct patterns of demyelination between H-2^b ^and H-2^b/s ^EAE mice. We provide evidence of preferential depletion of MAG, suggestive of oligodendrogliopathy, in concert with reduction of axonal NF160 and rise of PARPp85, in severely relapsing H-2^b/s ^mice. We propose that MOG-induced relapsing EAE in (B6 × SJL) F1 (H-2^b/s^) mice may serve as a useful model in studying mechanisms of autoimmune-induced preferential loss of MAG and its impact on oligodendroglial pathology.

## Methods

### Mice and induction of EAE

EAE was induced in 8–10-week-old C57BL/6 (B6) (H-2^b^) and (B6 × SJL) F1 (H-2^b/s^) female mice (Jackson Laboratories, Bar Harbor, Maine) by immunization with MOG_35–55 _(MEVGWYRSPFSRVVHLYRNGK), 99% pure by HPLC, (Caltech, Pasadena, CA), as previously described (8). Mice were daily observed for clinical symptoms of EAE. The clinical grade was scored as follows: 0.5, partial loss of tail tonicity; 1, complete loss of tail tonicity; 2, flaccid tail and abnormal gait; 3, hind leg paralysis; 4, hind leg paralysis with hind body paresis; 5, hind and foreleg paralysis, 6, moribund.

### Antibodies

The following primary antibodies were used: monoclonal anti-MAG (1:500 – Chemicon Intl Tamecula CA); and anti-NF160 (1:320 – Sigma); polyclonal anti-MOG (1:500), anti- MBP (1:500), and anti-GAPDH (1:1000 – Santa Cruz Biotechnology, Santa Cruz, CA); anti-PARPp85 (1:500 – Promega, Maddison, WI). Secondary antibodies were peroxidase-conjugated and directed against, mouse, goat or rabbit IgG (R&D Systems, Minneapolis, MN).

### Sample preparation and western blot

Mice were sacrificed at different stages of relapsing-remitting disease: acute, remission, relapse and chronic. Approximately 6–7 mice from each strain were analyzed at each stage of disease, except for H-2^b ^relapsing and H-2^b/s ^chronic sustained, where 3–4 mice were examined (Table 1). Anaesthetized mice were perfused through the heart with 10 ml of ice-cold Phosphate buffered saline (PBS), and 5 mm thick tissue blocks were dissected from cervical, thoracic and lumbar spinal cord. Tissue blocks were either embedded in Tissue Compound (Sigma, St Louis, Missouri) for tissue section preparation, or alternatively snap frozen, for protein isolation. All tissue samples were stored at -70°C until use.

**Table 1:** Disease Incidence and Mean Clinical Severity Score in H-2^b/s ^and H-2^b ^Mice

**Disease Incidence and Mean Clinical Severity Score**						
	**Acute**		**Relapse**		**Chronic Sustained**	
			
**Strain**	Severity	Incidence	Severity	Incidence	Severity	Incidence

H-2^b/s^	3.8 ± 0.5	18/18	2.9 ± 0.05	9/12	2.2 ± 0.05	3/12
H-2^b^	1.7 ± 0.3	18/18	1.0 ± 0.1	3/12	NA	0/12

Protein was isolated from fresh frozen spinal cord tissue using TRIzol reagent (Gibco BRL, Grand Island, NY) according to the procedure recommended by the manufacturer. Equal amounts of protein (approximately 20 μg/lane) from each sample were loaded per lane for Western blot analysis. Samples were loaded at non-reducing conditions onto NuPage Novex Tris-Acetate Gels (Invitrogen, Carrsbad, CA), resolved by electrophoresis. Electrophoresed proteins were then transferred from the gel onto nitrocellulose membrane. Membrane was cut into 3–4 strips, each containing proteins of different molecular weight. Each membrane strip was probed separately with the appropriate primary antibody overnight at 4°C, washed three times with 0.1% Tween 20- Tris-buffered saline, and then incubated with peroxidase conjugated secondary antibody. The membrane bound peroxidase activity was detected using ECL Plus western blotting detection kits (Amersham, Arlington Heights, IL). Chemiluminescent images were captured and analyzed by a Kodak Digital Science Image Station 440CF. All blots were analyzed within the linear range of exposure. In each sample levels of MAG, MOG, MBP, NF160 and PARPp85 were normalized by corresponding levels of GAPDH.

### Percoll gradient separation of glia and inflammatory cells

In three separate experiments, which included a total of 10 mice, we used discontinuous Percoll gradients to separate infiltrating lymphocytes from resident spinal cord cells. In each experiment, spinal cord tissue was pooled from 3–4 mice with relapsing EAE. Anaesthetized mice were perfused through the heart with 20 ml of ice-cold phosphate buffered saline (PBS) (Sigma, St Louis, Missouri), and spinal cord was dissected. Tissue was teased through a wire mesh, and the cell suspension was treated with DNAse (10 μg/ml) for 10 min at 37°C. DNAse treated tissue was then overlaid on a discontinuous Percoll gradient (Sigma, Saint Louis, Missouri), and centrifuged at 3000 rpm for 20 minutes. Infiltrating lymphocytes were collected in the interface between 25% and 55% Percoll, while remaining the resident cells, including glia and neurons, separated on the top of the Percoll gradient.

### Two-color immunostaining and confocal microscopy

Frozen sections (8 – 10 μM) were used to analyze phenotypes of infiltrating cells by immunofluorescence, following a routine procedure [8]. Briefly, sections were air-dried, acetone-fixed, and treated with 10% normal donkey serum for 10 minutes, followed by overnight incubation with relevant primary antibodies (1: 100), in a moist chamber at +4°C. The slides were then washed and incubated with secondary fluorochrome labeled antibodies (1:200), (Santa Cruz Biotechology, CA) for 30 minutes. Nuclear staining with 30 nM 4',6-diamidino-2-phenylindole, dihydrochlpride (DAPI) (Molecular Probes). After washing, the slides were mounted in Gelmount (Biomeda, Foster City, CA) and analyzed by light and fluorescent microscopy. Images were captured on a Nikon Eclipse 600 epifluorescent microscope with a Princeton Instruments Micromax 5 MHz cooled CCD camera.

### Hematoxylin-eosin (H&E) staining

Mononuclear cell infiltration was visualized by H& E staining of a 10 μM thick frozen sections [8]. Briefly, H& E staining sections were air-dried, fixed in 10% formaldehyde, dehydrated, stained for 1 minute with hematoxylin, then for 2 minutes with eosin (Sigma, St Louis, Missouri), then dehydrated, and mounted in Permount (Fisher Scientific, Pittsburgh, PA). Luxol fast blue was dropped on the air-dried section, heated at 60°C for two minutes, washed, dehydrated, and mounted in Permount.

### Statistical analysis

The significance of differences between the groups was calculated by the Student's t-test or paired-test as appropriate. The level of statistical significance was set at ninety-five percent.

## Results

### Most profound depletion of MAG levels was exclusive for H-2^b/s ^relapsing mice

**Figure 1:**
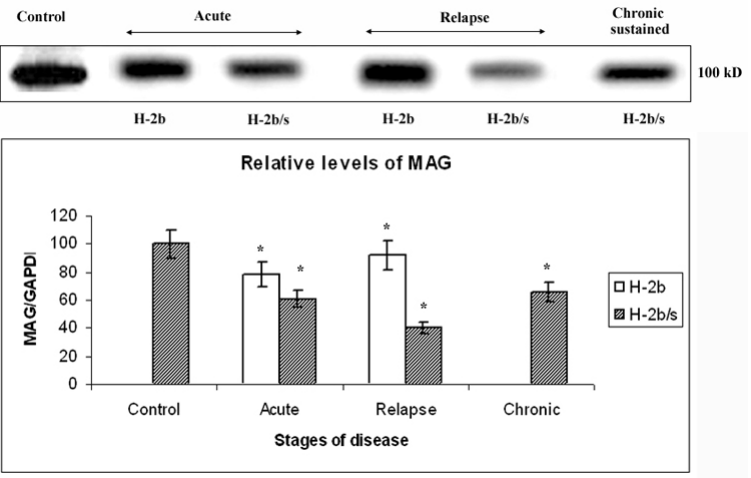
**Profound depletion of MAG levels occurs in relapsing H-2^b/s ^mice**. Dissimilar regulation of relative levels of MAG was observed between H-2^b ^and H-2^b/s ^mice throughout EAE. Levels of MAG in spinal cord of EAE mice were measured by western blot. Approximately 20 μg of total protein was loaded per lane. Total of 3–4 mice from H-2^b/s ^and H-2^b ^strains with acute and relapsing stages of the disease were analyzed. Only H-2^b/s ^mice (3 mice) with chronic-sustained disease were examined. Representative blot of 3–4 similar experiments shows: acute – H-2^b/s ^(13 dpi, grade 4.5), H-2^b ^(16 dpi, grade 4); relapse – H-2^b/s ^(21 dpi, grade 3.5, 1^st ^relapse), H-2^b ^(27 dpi, grade 2, 1^st ^relapse); and chronic-sustained – H-2^b/s ^(72 dpi, grade 2.5). Relative levels of MAG were calculated as ration to corresponding levels of GAPDH (see Fig. 4). Compared to levels in control and in H-2^b ^mice, H-2^b/s ^mice markedly reduced their relative levels of MAG during acute and chronic. Most profound depletion of MAG was found in relapsing H-2^b/s ^mice. Corresponding levels of GAPDH are shown in Fig. 6. Membrane was stripped and re-probed with MOG, MBP, NF160 and PARPp85 antibodies (Fig. 2, 4, 6 and 7A).

In spinal cord tissue of EAE H-2^b/s ^and H-2^b ^mice, we assessed specific changes of relative levels of myelin-associated glycoprotein (MAG) by western analysis (Fig. 1). During acute, relapsing and chronic-sustained disease, relative levels of MAG were measured and compared to control values. Control levels of MAG, arbitrarily taken as 100, were indistinguishable between the two strains of mice. During acute disease, relative levels of MAG were markedly reduced in H-2^b/s ^compared to control (61 ± 5.9 vs. 100; p < 0.05) and to H-2^b ^mice (61 ± 5.9 vs. 79; p < 0.05). H-2^b/s ^mice reduced their content of MAG approximately 40% compared to control and about 20% compared to H-2^b ^mice with acute disease. During EAE relapse, differences in content of MAG became more pronounced between the two strains of mice. In relapsing H-2^b/s ^mice, levels of MAG dropped more than two fold compared to control values (40 ± 3.7 vs. 100; p < 0.05), while content of MAG in relapsing H-2^b ^mice did not change significantly (92 ± 8.8 vs. 100). Loss of MAG in H-2^b/s ^mice with chronic-sustained disease, resembled that in acute, (66 ± 6.3 vs. 100; p < 0.05). Overall, while both strains significantly depleted their levels of MAG during acute disease, loss of MAG in H-2^b/s ^mice was approximately 20% higher then in H-2^b ^mice. Dissimilar to the shared trend in reducing MAG levels by both strains of mice during acute disease, sharp profound drop of MAG was found only in H-2^b/s ^strain of mice during EAE relapse. H-2^b/s ^mice readily exhibited approximately three times more frequent, and more severe relapsing disease compared to H-2^b ^mice (2.9 ± 0.05 vs. 1.0 ± 0.1 – Table 1).

### MOG levels were markedly lower in H-2^b/s ^compared to H-2^b ^mice with acute disease, while MOG is being relatively spared during relapse

**Figure 2:**
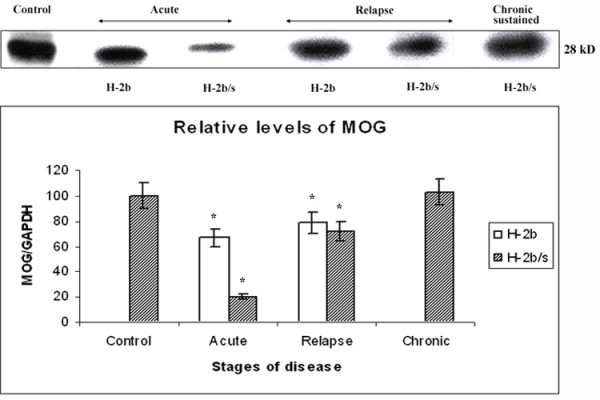
**Distinct patterns of MOG reduction between H-2^b/s ^and H-2^b ^mice throughout disease**. Distinct patterns of MOG-specific demyelination were observed between H-2^b ^and H-2^b/s ^mice throughout EAE. Relative levels of MOG were measured similarly as levels of MAG (Fig.1). As opposed to regulation of MAG, most profound depletion of MOG was observed in H-2^b/s ^mice during acute EAE. Lesser then in H-2^b/s^, loss of MOG was noted in acute H-2^b ^mice during acute disease. Loss of MOG, lesser then in acute but significant compared to control values, was equally found in H-2^b ^and H-2^b/s ^mice with relapsing disease. In H-2^b/s ^mice with chronic-sustained disease levels of MOG were comparable to control values. Stripped and reprobed membrane was shown. Corresponding levels of GAPDH are shown in Fig. 6.

**Figure 3:**
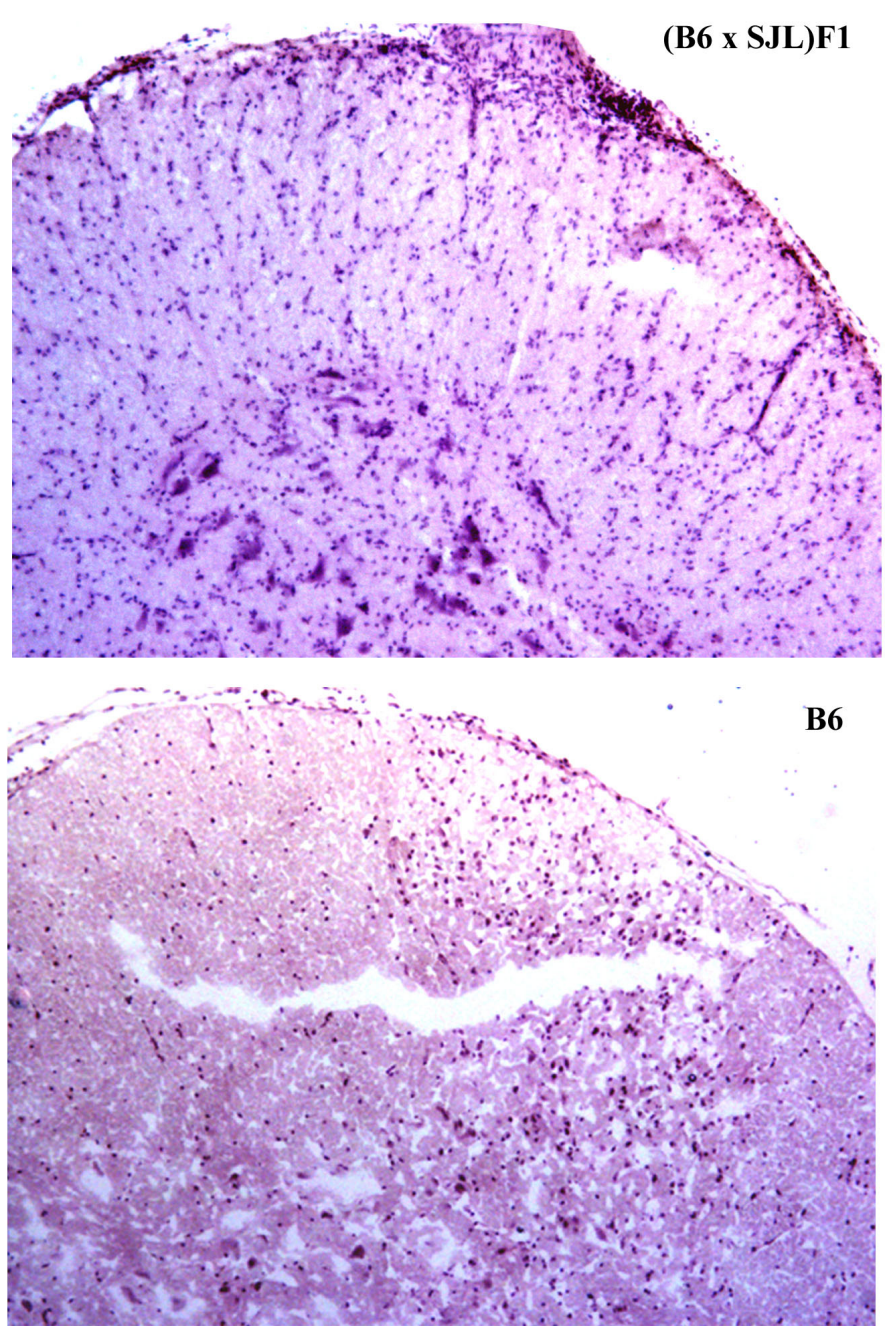
H&E staining reveals an extensive mononuclear cell infiltration in spinal cord of (B6 × SJL) F1 (H-2^b/s^) relapsing mice (top panel). In these mice inflammatory cells are evident throughout the spinal cord parenchyma. Lesser degree of inflammation was observed in relapsing B6 (H-2^b^) mice (lower panel). Representative image shows lumbar spinal cord from relapsing H-2^b/s ^mouse, with clinical severity score of 3, and relapsing H-2^b ^mouse with severity score of 2. (H&E × 10).

Similarly to regulation of MAG, in acute disease, both strains of mice exhibited depletion of MOG compared to control values, H-2^b ^(67 ± 6.5 vs. 100; p < 0.05) and H-2^b/s ^mice (20 ± 1.8 vs. 100) (Fig. 2). During acute EAE, H-2^b/s ^mice, which compared to H-2^b ^readily developed more severe clinical disease (3.8 ± 0.5 vs. 1.7 ± 0.3 – Table 1), reduction of MOG levels was over three fold more pronounced than in H-2^b^. Dissimilar to sharp differences between the two strains of mice in MOG loss during acute EAE, in relapsing disease, loss of MOG was comparable between strains. In relapsing mice, significant depletion of MOG over control levels was in H-2^b ^mice (79 ± 7.4 vs. 100; p < 0.05) and in H-2^b ^mice (72 ± 6.9 vs. 100; p < 0.05). Compared to reduction of MOG in acute disease, corresponding levels in relapsing H-2^b ^mice did not significantly change (67 ± 6.5 vs. 79 ± 7.4). On the contrary, compared to acute levels of MOG in H-2^b/s ^mice, MOG was markedly preserved during EAE relapse (20 ± 1.8 vs. 72 ± 6.9; p < 0.05). Interestingly, more profound mononuclear cell infiltration was readily observed throughout the spinal cord of relapsing (B6 × SJL) F1 (H-2^b/s^), compared to relapsing B6 (H-2^b^) mice (Fig. 3). Concordant with relative preservation of MOG during relapsing disease, in H-2^b/s ^mice with chronic sustained disease this content did not appear to differ from control values (103 ± 10.2 vs. 100).

### Modest reduction of relative levels of MBP was found only in H-2^b/s ^mice with relapsing and chronic disease

**Figure 4:**
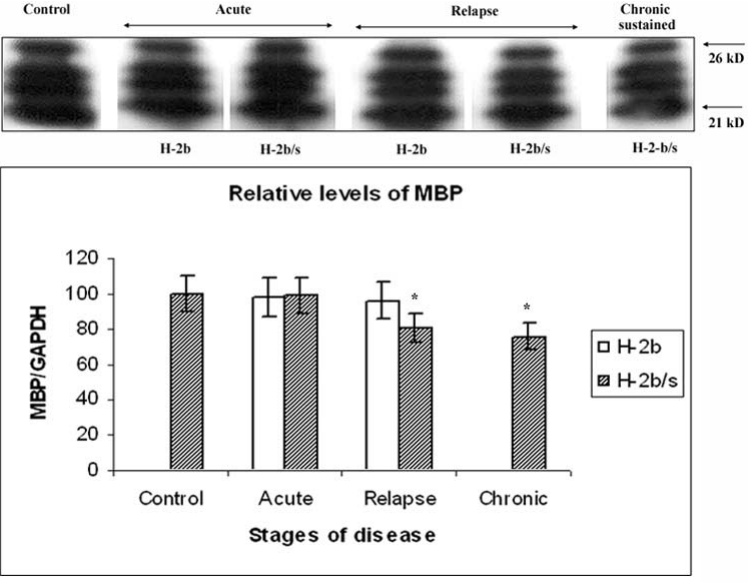
**Discrete changes in MBP levels in CNS of H-2^b/s ^and H-2^b ^mice throughout EAE**. Discrete changes in MBP levels were observed in spinal cord of H-2^b ^and H-2^b/s ^mice throughout EAE. Relative levels of MBP were measured similarly as levels of MAG (Fig. 1). Compared to control values, modest reduction of MBP levels was measured only in H-2^b/s ^mice during relapsing and chronic-sustained EAE. This antibody revealed presence of four distinct closely related 21–26 KD isoforms of MBP in control and EAE spinal cord, similarly as reported by the manufacturer of this antibody. These isoforms may be the result of posttranslational modification of MBP. Stripped and re-probed membrane was shown. Corresponding levels of GAPDH are shown in Fig. 6.

Similarly to constitutive levels of MAG and MOG, control levels of MBP did not differ significantly between the strains. In H-2^b ^mice with acute and chronic disease, MBP levels remained close to control values (98 ± 1.0 and 96 ± 0.9 vs. 100 respectively) (Fig. 4). Similarly to situation in H-2^b ^mice with acute disease, H-2^b/s ^mice did not exhibit notable changes in their relative levels of MBP during acute EAE (99 ± 9.7 vs. 100). As opposed to relative preservation of MBP during acute disease, in H-2^b/s ^mice with relapsing and chronic disease modest reduction of MBP was found (81 ± 7.7 and 76 ± 7.7 vs. 100; p < 0.05).

**Figure 5:**
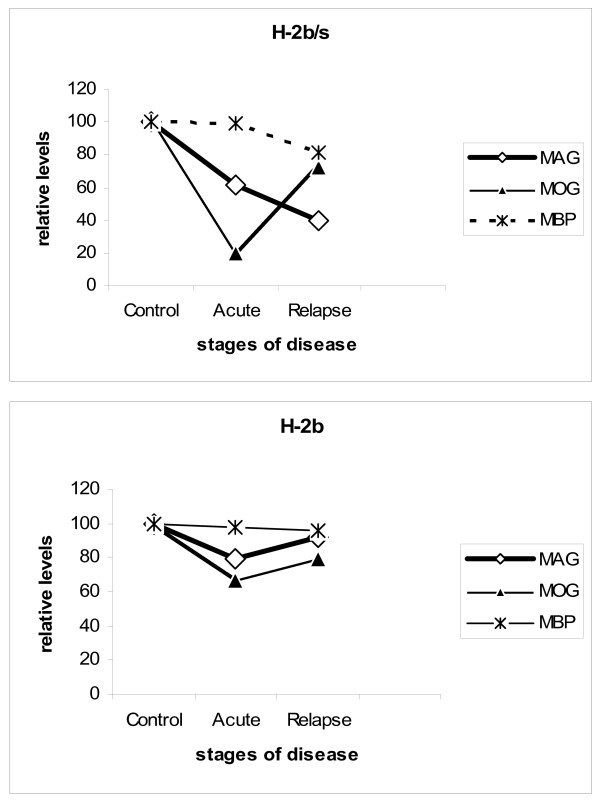
**Correlation between levels of MAG, MOG and MBP **inH-2^b/s ^and H-2^b ^mice has revealed dissimilar regulation of MAG and MOG in relapsing H-2^b/s ^mice.

Interestingly, levels of all three examined myelin proteins, MAG, MOG and MBP displayed distinct regulatory patterns in regard to course of disease and to strain of mice (Fig. 5). In acute disease, both MOG and MAG levels were depleted in both strains of mice. However, specific loss of MOG exceeded that of MAG and loss of both proteins in H-2^b/s ^was markedly higher then in H-2^b ^strain in acute EAE. Conversely, during relapse, levels of MAG had dropped markedly only in H-2^b/s ^mice, while remaining close to control values in relapsing H-2^b ^mice. Levels of MOG raised compared to acute disease and were comparable between two strains during relapsing EAE. Modest reduction of MBP levels was found only in H-2^b/s ^mice with relapsing and even more pronounced during chronic disease.

### Reduction of axonal neurofilament medium chain (NF160) correlates with depletion of relative levels of MAG in relapsing H-2^b/s ^mice

Profound loss of MAG during acute and especially during relapsing EAE in H-2^b/s ^mice (Fig. 1) is suggestive of autoimmune mediated dysfunction and/or even apoptosis of oligodendrocytes. Proper function of oligodendrocytes is essential for maintenance of axonal homeostasis. To further investigate if axonal dysfunction and/or apoptosis accompany this profound specific depletion of MAG, we measured levels of neurofilament medium chain (NF160), and levels of a cleaved fragment of DNA binding protein PARP, accordingly.

**Figure 6:**
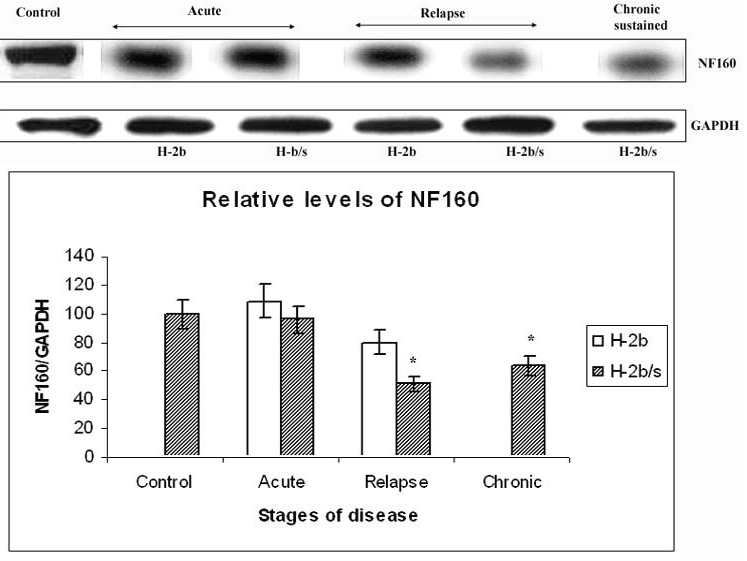
**Distinctive changes of NF160 levels in CNS throughout disease between H-2^b/s ^and H-2^b ^mice**. Relative levels of medium chain of neurofilament (NF160) were significantly depleted in mice with relapsing and chronic disease. Relative levels of NF160 were measured similarly as levels of MAG (Fig.1). Most profound drop in NF160 levels was observed in relapsing H-2^b/s ^mice.

Reduction in axonal neurofilament medium chain (NF160) was not observed during acute disease in either H-2^b ^or H-2^b/s ^strain of mice (Fig. 6). Conversely, mice with relapsing disease exhibited changes in axonal NF160 content. In relapsing H-2^b/s ^mice, NF160 levels were approximately two fold reduced compared to control (51 ± 4.9 vs. 100; p < 0.05). Reduction in levels of NF160 in H-2^b ^mice was less prominent compared to H-2^b/s ^mice but still significant compared to control (80 ± 7.7; p < 0.05). In H-2^b/s ^mice with chronic sustained disease, relative levels of NF160 were slightly higher than in relapsing stage of disease, but still markedly lower compared to control (64 ± 6.1 vs. 100; p < 0.05).

### Sharp elevation of PARP-p85 levels was found in relapsing H-2^b/s ^mice

**Figure 7:**
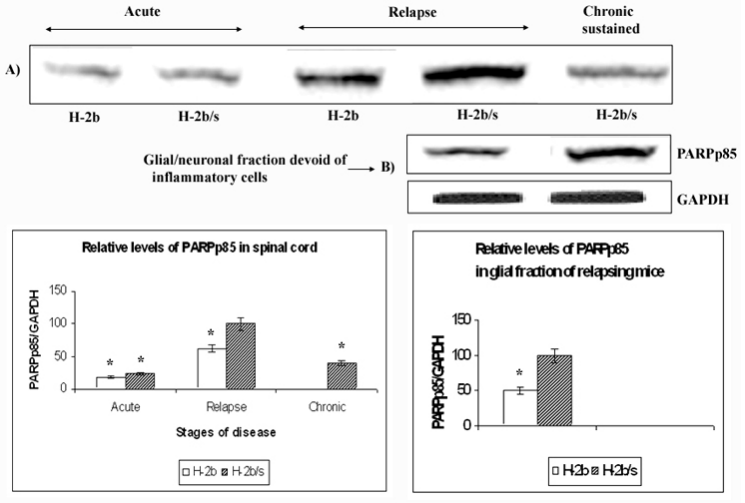
**Differences in levels of PARPp85 between H-2^b/s ^and H-2^b ^EAE mice**. Relative levels of PARPp85 were measured in spinal cord (A) and in Percol gradient separated fraction of spinal cord, which was devoid of infiltrating inflammatory cells (B). Relative levels of PARPp85 in spinal cord were determined similarly as levels of MAG (Fig. 1). The highest level of PARPp85 was found in relapsing H-2^b/s ^mice, and this value was arbitrarily taken as 100. Compared to this highest level, significantly lower relative levels of PARPp85 were observed during acute and chronic disease. Stripped and re-probed membrane was shown for levels of PARPp85 in spinal cord. Corresponding levels of GAPDH are shown in Fig. 6.

Caspase-cleaved fragment of poly (ADP-ribose) polymerase (PARPp85) was not observed in control spinal cord tissue of either strain of mice (not shown). In acute disease, small amounts of PARPp85 (18 ± 1.5 in H-2^b^, and 24 ± 2.2 in H-2^b/s^) were measured (Fig. 7A). Compared to acute disease, sharp elevation of relative levels of PARPp85 was found in H-2^b/s ^strain during EAE relapse. This highest level of PARPp85 was arbitrarily taken as 100. In relapsing H-2^b ^mice, levels of PARPp85 were almost two fold less elevated then in relapsing H-2^b/s ^mice (62 ± 6.3 vs. 100: p < 0.05). The sudden rise in PARPp85 in CNS of relapsing mice indicated apoptosis of either activated inflammatory cells and/or resident glia. To investigate the contribution of non-inflammatory cell phenotypes to the abrupt elevation of cleaved PARP in relapsing mice, we measured PARPp85 in glial/neuronal fraction following Percoll separation (Fig. 7B). Similar to whole spinal cord tissue, in the glial/neuronal fraction, elevation of PARPp85 was similar when H-2^b ^and H-2^b/s ^strains were compared (50 ± 4.8 vs. 100; p < 0.05). However, when glial and whole tissue PARPp85 levels were compared for each strain of mice, it became apparent that the glial/neuronal fraction represented approximately 60% in H-2^b ^and 75% in H-2^b/s ^mice.

**Figure 8:**
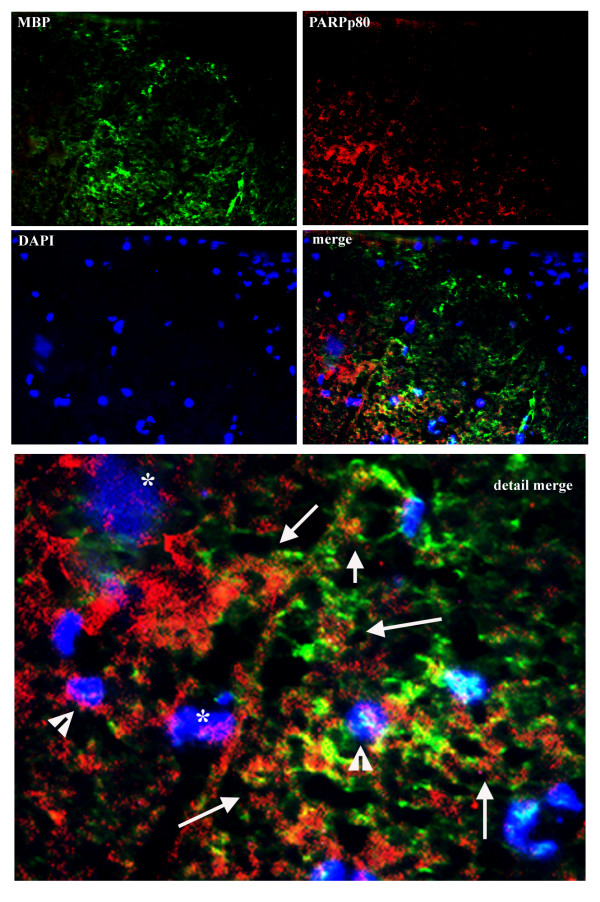
Two-color immunostaining reveals co-localization of PARPp85 and some MBP immunoreactivity (merge and merge detail). Some PARPp85 is found in cell nuclei, whose morphology is suggestive of apoptosis (asterix). Other nuclei with PARPp85 immunoreactivity appear round and small (arrowhead). The remaining PARPp85 immunostaining appears extranuclear and relates to cells expressing MBP immunoreactivity (arrows). Note relative absence of PARPp85 in submeningeal mononuclear cells (merge – upper right). (Two-color immunofluorescence × 20 and 40).

As anticipated, appreciable levels of PARPp85 were measured in the fraction devoid of inflammatory mononuclear cells, thus suggesting that cleavage of PARP occurs in glia and/or neurons during EAE relapse. This observation was further confirmed by immunostaining. By two-color immunostaining it became apparent that some of PARPp85 immunoreactivity co-localized with some MBP containing cells in spinal cord of relapsing H-2^b/s ^mice (Fig. 8). While some of the PARPp85 immunostaining was observed within the cytoplasm, more importantly it was also found in some nuclei (Fig. 8). Consistent with finding of PARp85 in percoll gradient-separated glial/neuronal fraction, PARPp85 immunostaining did not notably co-localize to infiltrating submeningeal mononuclear cells (Fig. 8, merge – upper right).

**Figure 9:**
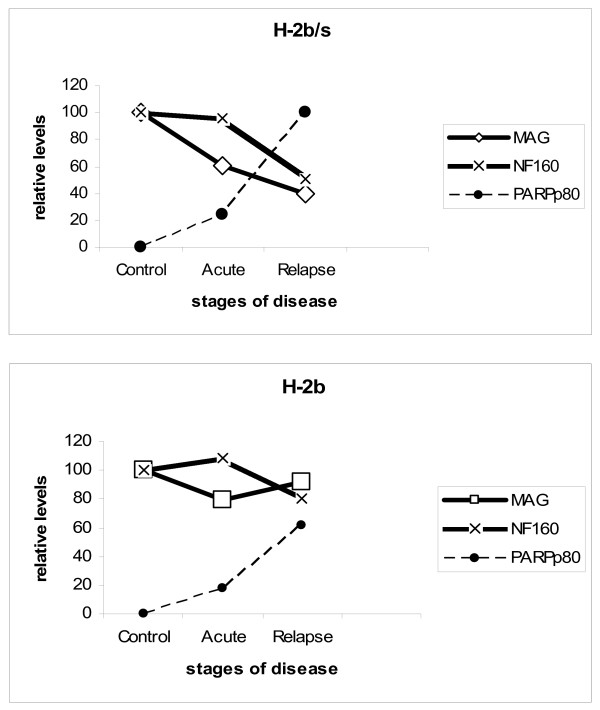
**Correlation between MAG, NF160 and PARPp85 levels **in H-2^b/s ^and H-2^b ^mice has revealed similar regulation of MAG and NF160 in relapsing H-2^b/s ^mice. Dissimilar to profound drop of MAG and NF160, sharp elevation of PARPp85 was observed in these mice.

Taken together, profound reduction of NF160, accompanied with sudden elevation of PARPp80 and marked reduction of MAG was found in relapsing H-2^b/s ^mice. (Fig. 9)

## Discussion

Oligodendroglial damage and/or dysfunction are critical features in the pathogenesis of MS [3]. Damage to oligodendrocytes precludes maintenance of homeostatic myelin-axonal signaling and their proper communication. Those changes are typically thought to be irreversible and to have profound impacts on function of myelinated axons [11]. Distal oligodendrogliopathy, whose pathogenetic mechanisms are still unclear, is readily found in the subtype III of acute MS demyelinating lesion [2,12]. It is unknown if there is an appropriate EAE correlate for this specific subtype of MS immunopathology. Finding an EAE model which recapitulates at least some key features observed in the subtype III MS lesion, will greatly propel studies on the mechanisms leading to oligodendroglial damage and enable development of new therapies. While such model is desirable one should always remain critical about strain specific differences in regulation of autoimmune responses between rodents and humans. Nevertheless, genetic regulation of immune responses makes some strains of mice more comparable to some aspects of human disease than others. We reported distinct appearance and distribution of demyelinating lesions in spinal cord of H-2^b ^and H-2^b/s ^EAE mice, by means of histochemistry. While in H-2^b ^mice demyelinating lesions were found predominantly in submeningeal areas, in H-2^b/s ^mice, small, scattered, perivenular lesions were observed throughout white matter parenchyma (Fig. 3) and [8]. Those differences were suggestive of distinctive genetic regulation of autoimmune mediated demyelination between H-2^b ^and H-2^b/s ^EAE mice. Molecular mechanisms leading to higher susceptibility of hybrid compared to parental strain of mice remain elusive. Based on this initial observation, we sought to answer if those apparently discrete autoimmune responses, initiated by immunization with MOG_35–55_, cause distinctive damage to myelin and/or the myelin forming cells, oligodendrocytes, which then underlie readily relapsing and more severe clinical disease in H-2^b/s ^mice. Therefore, in this study we measured and compared changes in relative levels of the myelin proteins MOG, MAG and MBP, throughout disease, in these two genetically related strains of mice. We anticipated that autoimmune mechanisms governed by T-cells and antibodies initiated towards MOG_35–55_, will be primarily responsible for an initial depletion of MOG levels, in both strains of mice. Our data confirmed marked depletion of MOG levels during an acute disease in both H-2^b ^and H-2^b/s ^mice. However, relative levels of MOG are more profoundly depleted in H-2^b/s^, which developed significantly more severe acute disease compared to H-2^b ^mice. More pronounced loss of MOG, found in H-2^b/s ^mice with acute and relapsing disease, was in concert with elevated memory T cell and antibody responses to MOG_35–55 _in these mice [8,10]. Interestingly, depletion of MOG was less apparent in relapsing mice compared to acute, in both strains. Recovered levels of MOG in relapsing mice may result from attempted remyelination, which has been reported to take place in MOG induced EAE in H-2^b ^mice by our laboratory and others [13,14].

Compared to MOG, which was an apparent target of autoimmune-induced responses, and which is located in the outer myelin wraps, MAG was an unlikely candidate for autoimmune-induced damage. This myelin protein is located in the most inner loop of the myelin sheet and it serves as a specific oligodendroglial functional marker [11]. Specific periaxonal localization of MAG implicates a role of this glycoprotein in bi-directional oligo-axonal communication. It is proposed that MAG serves as a ligand for a receptor complex involving the Nogo receptor and/or gangliosides containing 2,3-linked sialic acid. It provides signal necessary for the maintenance of myelinated axons and acts as one of white matter inhibitors of neurite outgrowth. Conversely, MAG serves as a receptor for axonal signals that regulate homeostasis and survival of oligodendrocytes [15]. CNS pathologies, including autoimmune myelin specific mechanisms, inflammation, reduced vascular perfusion, can induce oligodendroglial dysfunction and/or apoptosis mediated death of oligodendrocytes. In those conditions, reduction in specific levels of MAG appeared either as an early indicator of compromised oligodendroglial function, or as a sign of oligodendrogliopathy [16]. Demyelination leads to axonal dysmorphia and degeneration in MS and other demyelinating diseases. A unifying hypothesis that links neuronal stress associated with demyelination-induced axonal dysfunction to immune recognition and immunopathology has been recently proposed [17].

Our findings of preferential loss of MAG over MBP correlate well with observation based on immunocytochemical distribution of MAG and MBP in MS lesions [18]. The authors reported that the most striking finding in MS lesions was the extent of MAG depletion into white matter that appeared normal by criteria of MBP immunostaining. They also described that loss of MAG had extended far beyond the margin of acute demyelination, where MBP staining of degenerated sheaths was often increased. They suggested that periaxonal MAG is altered before the myelin breakdown begins in MS. We hypothesize that in H-2^b/s ^EAE mice autoimmune induced stripping of MOG from the myelin sheath, and subsequent autoimmune-induced inflammation cause changes in axonal to oligodendrocyte signaling through MAG, with subsequent reduction of MAG levels. Accordingly, in H-2^b/s^mice we found markedly reduced levels of MAG in acute and even more pronounced in relapsing disease. In addition inflammatory mediators induce a cascade activation of resident glial cells, microglia, astrocytes, dendritic cells, which extends beyond margins of demyelinating lesion. It is possible that in normal appearing white matter MAG becomes depleted as a result of neighboring cell activation and subsequent expression and local release of inflammatory mediators. Such mechanism may be responsible for even more profound loss of MAG found in H-2^b/s ^mice with relapsing and chronic sustained disease.

It is also possible that some other genetically determined mechanism, which may or may not be directly related to autoimmune response to MOG in H-2^b/s^mice underlies this profound loss of MAG. Interestingly, in patients with relapsing and remitting MS, who died shortly or during the onset of relapse, oligodendroglial apoptosis and microglial activation without apparent demyelination or extensive adjacent inflammation were observed [19]. While both studies in MS [[18] and [19]] used classical histopathology and immunopathology approaches, our study, similarly to some other studies in EAE [20] utilizes biochemical measurement of molecular markers of demyelination. Our ongoing histopathology studies of these and other markers depicting demyelination, apoptosis and oligo-axonal communication, will enable better comparison of biochemical changes found in relapsing H-2^b/s ^mice with histopathology changes reported in MS.

We anticipated that additional loss of other, more accessible myelin proteins, such as MBP, may occur during relapsing disease as a result of endogenous self priming and subsequent epitope spreading, at least in H-2^b/s ^mice. It is proposed that posttranslational modifications of MBP, such as citrulination or deamination, change binding affinity of this myelin protein for negatively charged lipids on the cytosolic portion of oligodendrocyte membrane thus making it relatively exposed and accessible to autoimmune attack [21]. Surprisingly, we did not find appreciable reductions in levels of MBP, in either low-relapsing H-2^b ^or severely relapsing H-2^b/s ^strains of mice. It is possible that relative lack of epitope spreading to MBP specific epitopes was due to non-permissive or low-permissive H-2 haplotypes in these two strains of mice. Because of the H-2 restriction of autoimmune responses to myelin proteins, immunodominant epitopes of MBP were not likely to induce disease in H-2^b ^mice, while in H-2^b/s ^mice those responses are low following immunization with MBP [[22,23], and Skundric DS, Dai R Zhou W. Immune responses to H-2^s ^restricted encephalitogenic epitopes in H-2^b/s ^mice, unpublished]. Taken together, dissimilar regulation of major myelin proteins MOG, MAG and MBP, suggest that genetically conducive environment in H-2^b/s ^mice favors an excessive loss of MAG during relapsing disease. Data suggest that potential oligodendroglial dysfunction and/or damage may underlie severe relapsing disease in these mice. Interestingly, distinct regulation of another myelin protein DM20, a splice variant of proteolipid protein (PLP), has been observed between two distinct patterns of recovery and types of remyelination in PLP-induced EAE in mice [24].

Homeostatic properties of oligodendroglia span beyond production, maintenance and escheatment of axons of the central nervous system (CNS). Proper oligodendroglial-axonal communication is essential for axonal function. Axonal dysfunction and even more profoundly, axonal loss, which is responsible for motor deficits in MS and EAE, are inherent. Neurofilaments (NF), the major type of intermediate filaments in adult neurons, comprise three subunits: light (NFL; 68 kDa), medium (NFM; 160 kDa) and heavy (NFH; 200 kDa). Quantification of neurofilaments is routinely used in MS and EAE as a measure of neurodegeneration [20,25]. We already showed that increased phosphorylation of medium and heavy neurofilaments [NF (M+H) P] associates with increased inflammation in MS lesions [26]. Recent findings point out an important role of NF160 in outside-in myelin-axonal signaling essential for axonal homeostasis. Therefore depletion of this neurofilament causes axonal dysfunction, a more discrete change compared to axonal degeneration or axonal loss [6,27]. Similarly to our findings reversible axonal injury but not axonal loss was found to accompany inflammation in acute EAE. Moreover, in vitro destabilization of axonal microtubules was directly mediated by co-culture with encephalitogenic T cells. This led to transient axonal dysfunction [28]. In MS and EAE axonal loss may partially occur as a result of Wallerian degeneration (WD) and in that case it does not necessarily links to demyelination and damage of oligodendrocytes. To get insight into relationship between demyelination and axonal loss, we compared MBP and NF160 specific immunoreactivity in H-2^b/s ^and H-2^b ^mice. This strategy did not reveal significant differences between two strains. In both H-2^b/s ^[see Additional file 1] and H-2^b ^mice [see Additional file 2], lack of co-localization may be found as well as areas of co-localized MBP and NF160 immunostaining. While WD is present in complex pathology of MS and EAE, it is not believed to be the major cause of disability in these diseases. WD has been reported in most EAE models, not only MOG-related [29]. It can be nicely demonstrated in 1 μm toluidine blue stained thin section or by EM. We showed presence of WD in SJL and C57BL/Ka mice with EAE induced by immunization with MBP or adoptive transfer of MBP sensitized and in vitro stimulated MBP specific T cells [30,31]. In our subsequent manuscript, we demonstrated presence of WD in (B6 × SJL)F1 mice immunized with MOG, using EM [13]. We similarly, observed signs of WD in MOG immunized B6 mice (Skundric, unpublished).

In EAE, an autoimmune-induced infiltration by activated T cells and monocytes triggers cascade reaction of glial activation [32]. Conversely, in CNS microenvironment, activation-induced apoptosis of inflammatory cells and functional and morphological changes of glia, including apoptosis, control the overall tissue damage and outcome of inflammation [[33-35], and [36]]. Poly (ADP-Ribose) polymerase (PARP) is a nuclear enzyme with an important role for DNA repair. Through its interactions with NF-κB, PARP is involved in regulation of cellular inflammatory responses [37,38]. In an EAE model in marmosets, activation of PARP-1 was observed in endothelial cells, neurons and glia, including oligodendrocytes, within demyelinating plaques [39]. Although PARP is not indispensable for progression of apoptosis the cleavage of PARP contributes to the irreversibility of apoptosis.

Overall, our data show that profound reduction of MAG levels correspond to depletion of NF160, and sharp elevation of PARPp85 exclusively in relapsing H-2^b/s ^mice. These data correlates with our previously reported findings of markedly increased infiltration by CD4+ T cell, levels of pro-inflammatory cytokine IL-16, activated caspase-3, in relapsing H-2^b/s ^compared to H-2^b ^mice [40]. Concurrent with our previous data, we readily observed mononuclear infiltrates around small blood vessels throughout white matter parenchyma in relapsing H-2^b/s ^mice, as opposed to H-2^b ^mice, where mononuclear cells preferentially infiltrated to submeningeal spaces (Fig. 3), [8]. This characteristic perivascular location of inflammatory cells in relapsing H-2^b/s ^mice may be responsible for eventual vascular changes and/or hypoperfusion, which may result in the loss of MAG [16]. Studies focusing on specific changes in vascular perfusion in relapsing H-2^b/s ^mice are under way in our laboratory.

We propose that MOG-induced EAE in H-2^b/s ^mice may prove as a useful model in studying underlying autoimmune mechanisms which regulate preferential loss of MAG and potentially related oligodendroglial dysfunction/apoptosis.

## Conclusion

Taken together, our results show genetically controlled distinct patterns of MOG and MAG specific depletion, in MOG-peptide induced EAE in H-2^b ^and H-2^b/s ^mice. The data also suggest distinctive immune regulation of relapsing compared to acute disease. A specific profound depletion of MAG, concomitant with marked depletion of axonal NF160, and sharp elevation of PARPp85 levels, occurred exclusively in relapsing H-2^b/s ^mice. Our findings indicate concurrence of preferential depletion of MAG, axonal dysfunction and signs of irreversible apoptosis with severe relapsing disease in H-2^b/s ^mice. Autoimmune-induced preferential loss of MAG over other myelin proteins may be suggestive of oligodendroglial dysfunction/damage in relapsing H-2^b/s ^mice.

## List of abbreviations

GAPDH: glyceraldehyde 3-phosphate dehydrogenase; EAE: experimental autoimmune encephalomyelitis; MS: multiple sclerosis; MAG: myelin associated glycoprotein; MOG: myelin oligodendrocyte glycoprotein; MBP: myelin basic protein; NF: neurofilament; PARP: poly (ADP-ribose) polymerase.

## Competing interests

The authors declare that they have no competing interests.

## Authors' contributions

DSS conceived the design of the study, provided critical analysis of the data and wrote the manuscript. She participated in EAE induction and western blot experiments; data evaluation and presentation, VZ was instrumental in statistical analysis of data and critical reading of the manuscript, RD and WZ participated in EAE induction and evaluation, sampling and preparation of tissue and western blot analyses.

## Supplementary Material

Additional file 1 Two-color MBP and NF160 specific immunostaining in spinal cord of relapsing H-2^b/s ^mouse. Lack of co-localization between MBP and NF160 immunostaining may be found, as well as areas of co-localized immunostaining. Click here for file

Additional file 2 Two-color MBP and NF160 specific immunostaining in spinal cord of relapsing H-2^b ^mouse. Similarly to situation observed in H-2^b/s ^mouse, lack of co-localization, as well as areas of co-localized immunostaining may be found in relapsing H-2^b ^mouse. Note that NF160 immunostaining can not be well appreciated in merged image because of the bright MBP staining, although it is apparent when shown separately Click here for file
