# Supplementary figures and images for: Autoimmune-induced preferential depletion of myelin-associated glycoprotein (MAG) is genetically regulated in relapsing EAE (B6 × SJL) F1 mice
Source: Mol Neurodegener. 2008 Jun 9;3:7. doi: 10.1186/1750-1326-3-7 (PMC2459167; doi:10.1186/1750-1326-3-7)

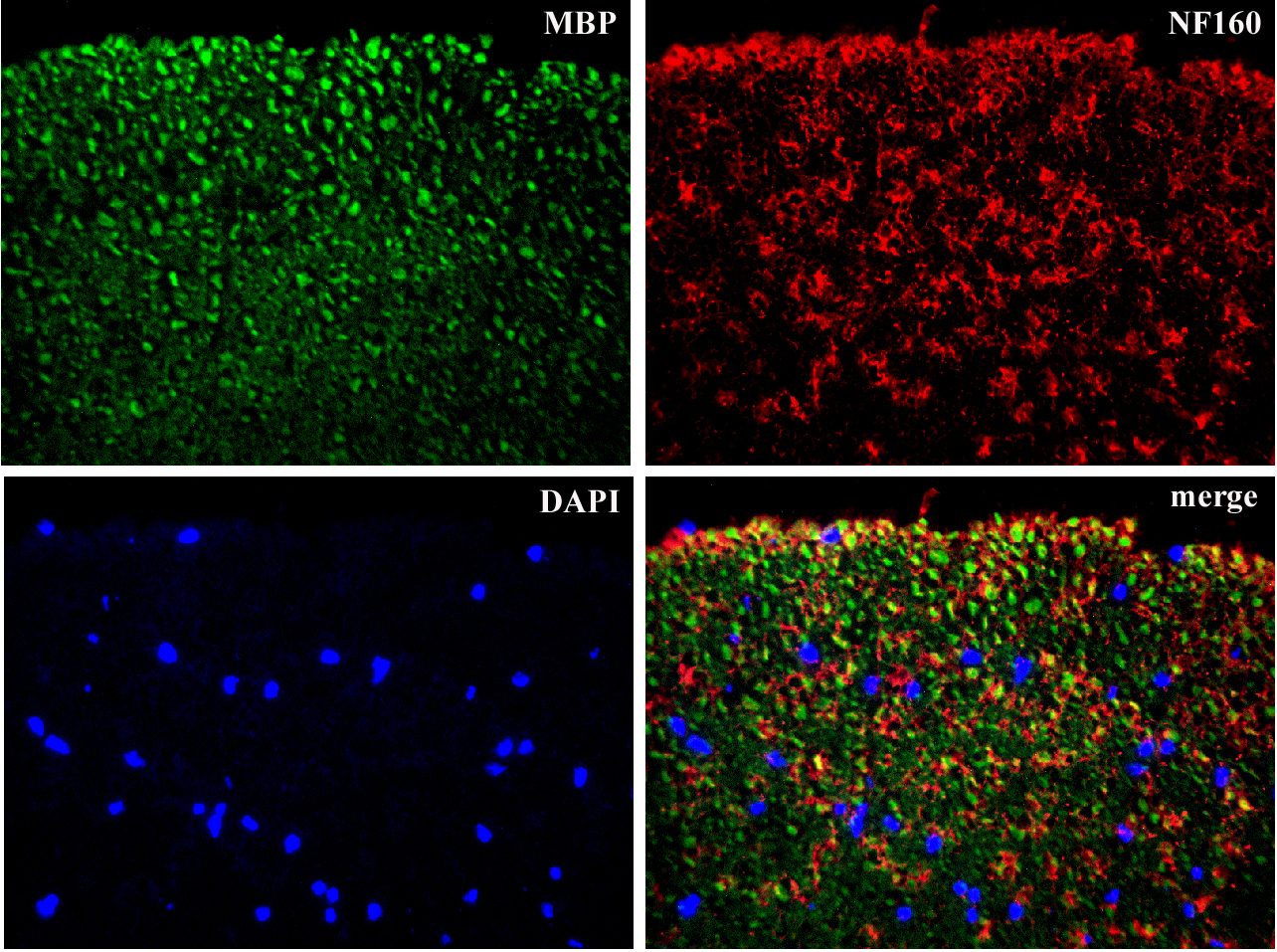

Supplement: Additional file 1 — Two-color MBP and NF160 specific immunostaining in spinal cord of relapsing H-2b/s mouse. Lack of co-localization between MBP and NF160 immunostaining may be found, as well as areas of co-localized immunostaining. [file 1750-1326-3-7-S1.jpeg]

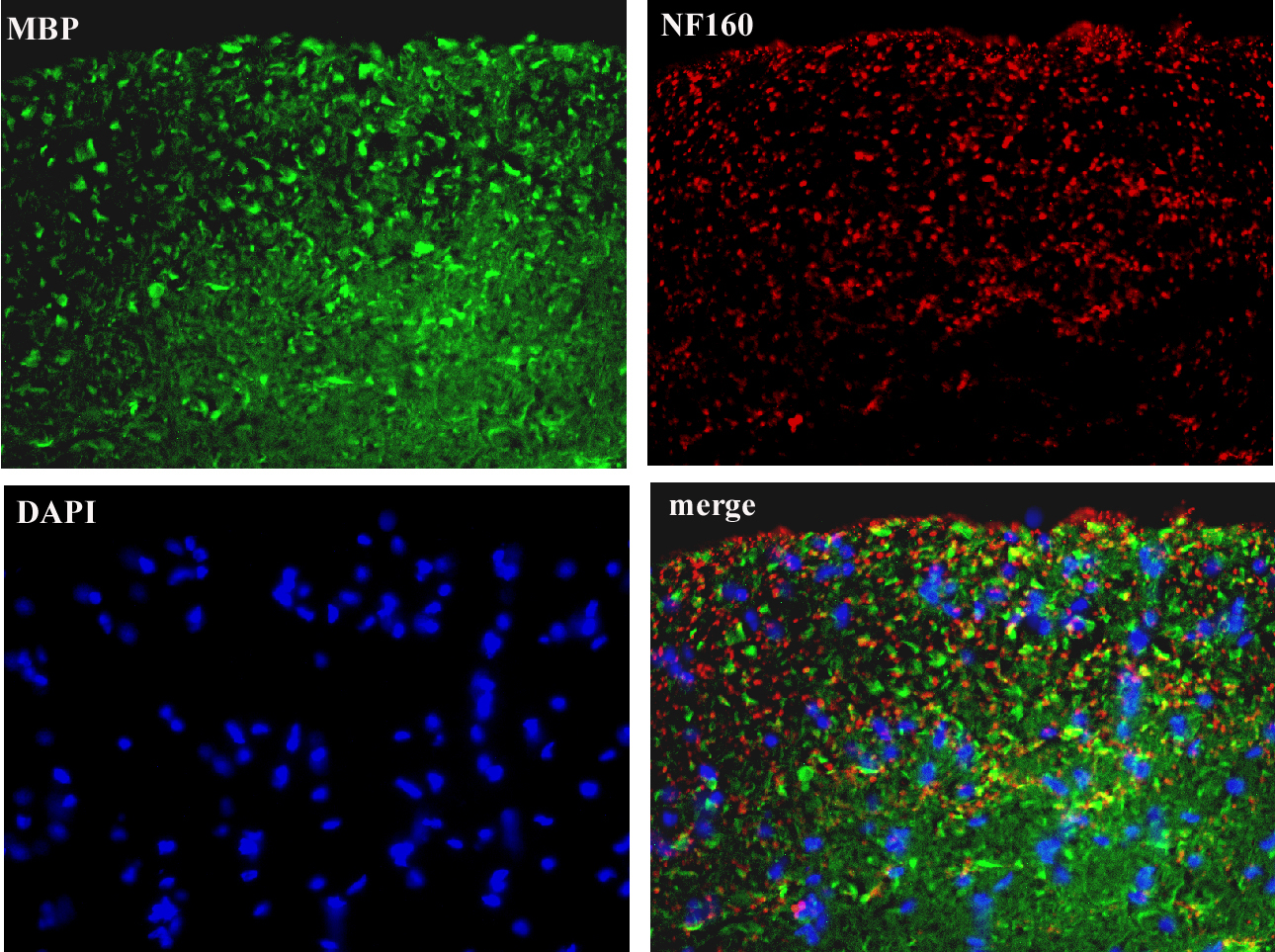

Supplement: Additional file 2 — Two-color MBP and NF160 specific immunostaining in spinal cord of relapsing H-2b mouse. Similarly to situation observed in H-2b/s mouse, lack of co-localization, as well as areas of co-localized immunostaining may be found in relapsing H-2b mouse. Note that NF160 immunostaining can not be well appreciated in merged image because of the bright MBP staining, although it is apparent when shown separately [file 1750-1326-3-7-S2.jpeg]
